# Supplementary material for: Chromothripsis during telomere crisis is independent of NHEJ, and consistent with a replicative origin
Source: Genome Res. 2019 May;29(5):737–49. doi: 10.1101/gr.240705.118 (PMC6499312; doi:10.1101/gr.240705.118)
Supplement: Supplemental Material [file supp_gr.240705.118_Supplemental_file_1.zip › contigs/annotated_contigs/DB48/contig.2.DB48_length_670_mean_cov_5.86268656716.docx]

**DB48_length_670_mean_cov_5.86268656716**

```
TCTCTATAATTTCAGCTCATAATTCTGATTTTTGTTAATACAAGAAATGTTTCTTCCTACATGTCTAATATACCTAGCCTGTTACCCCA
 >chr13:92238926-92239270 - E=3e-195
AATTTATTATAAACATATGTCTAAATGTATTTACATTTTTAATCGTAAGGTTGCATATCTACCAAATATTTCCATTTTGAATACTGAAA

CTAATCAAAAGTTAAAGAACTCTTCCCAAGACCACAAAATCAATATTGTGCAATATAACTCAAGACTTGTTGATATGGTTTGGCTCTGT

GTCCCCACCCAAATCTCATCTTGAATTATAATCCCCACATATCAAGAAAGGGACCTGCTGGGGGGTGATTGGATCAT|T|TTAAACAGT
                                                                               >chr13:9
AAAATGAAAAGAGAAAACCTCAGAAGACGTAGACAGAAGTTAATACACAGAGCTCAACTGCAAACTATGTGCATATTCTAATTTTTAAA
2237768-92238093 - E=9e-181
AGCATAACACACATAAGCAGTGGACAGTTCAGACAAACACTCACAAAAAGCACCATCACCAGAATCTTGGAAGACTTCTTTGTGGTCCT

TCTTGCAGCTGCTCCCTCCACCCCCCAAACACAACCACTATTTTGGGTCAAACGCCACAGATTTTTTTTGCATATTTTTGAGCTGCATA

TTAACTGTTAATATAATTGCACAGTATACCTCATTCCTTTTGCTCATCA
```
